# Fatty Acid Profile and Physicochemical Properties of *Moringa oleifera* Seed Oil Extracted at Different Temperatures

**DOI:** 10.3390/foods13172733

**Published:** 2024-08-28

**Authors:** Lourdes Cervera-Chiner, Sergio Pageo, Marisol Juan-Borrás, Francisco José García-Mares, María Luisa Castelló, María Dolores Ortolá

**Affiliations:** 1 Food Engineering Research Institute—FoodUPV, Universitat Politècnica de València, Camino de Vera s/n, 46022 Valencia, Spain; loucerch@upv.edu.es (L.C.-C.); spagdia@alumni.upv.es (S.P.); majuabor@upv.edu.es (M.J.-B.); mdortola@tal.upv.es (M.D.O.); 2 Department of Hydraulic Engineering and Environment, Universitat Politècnica de València, Camino de Vera s/n, 46022 Valencia, Spain; fjgarcia@gmf.upv.es

**Keywords:** moringa, oil, fatty acids, quality, color

## Abstract

*Moringa oleifera* Lam. (Moringaceae) is a tropical plant native to India. It is widespread throughout the southern hemisphere, with great adaptability to high temperatures and water scarcity. Its seeds have a great amount of oil with a high content of oleic acid, quite similar to olive oil. Therefore, this study is focused on the extraction of oil from moringa seeds via an automatic screw press extractor at different temperatures (70, 100, 130, 160, 190, and 220 °C) and on the analysis of its acidity, acid value, peroxide value (PV), saponification value (SV), iodine value (IV), optical properties, and fatty acids profile. The results showed that the oil yield was 19 ± 3% regardless of the temperature applied. The oil was stable from the oxidative point of view, with a high acidity. Temperature extraction did not significantly affect the SV and the IV. However, the extraction temperature should be below 190 °C to obtain a translucent and luminous oil with light yellow tones. The oil contains high levels of unsaturated fatty acids, especially oleic acid (ω9) (up to 77.8%) and linolenic acid (ω3) (3.4%). On the other hand, behenic (7%), palmitic (6%), stearic (5%), and arachidic (0.2%) were the dominant saturated acids. The good properties of moringa oil make it a good, sustainable alternative to vegetable oils.

## 1. Introduction

*Moringa oleifera* Lam. (Moringaceae) is a tropical tree belonging to the monogenic family *Moringaceae*, one of the best-known species. It is widely cultivated in countries of the southern hemisphere [1]. It is a tree native to the southern Himalayas and northeastern India, where 80% of its production is carried out [2]. Not only is it a vital crop in other Asian countries like Bangladesh, Afghanistan and Pakistan, to name but a few, but it is also widely developed in other areas of the world, such as South America, where it was introduced at the beginning of the 20th century, along with other Central American countries, such as Costa Rica and Nicaragua [3]. It should be noted that in Africa, there are some endemic species belonging to the northeast of the continent and Madagascar [4,5]. Therefore, warm and dry climates are the best conditions for the growth of this tree [3].

Moringa is a short-lived crop. It can live, at most, 20 years. It grows very quickly [4] and can reach heights up to 7–12 m [6]. It is characterized by its type of compound leaves, which can reach 60 cm in length, with a leaflet size length of 0.5 to 2 cm. Its fruits are formed in tricarperal pods of great length (between 20 and 45 cm) that contain 15 to 20 seeds that, during their growth, are green and have a fleshy body. Once they mature, they dry out, turning a brown hue [7].

*M. oleifera* is highly adaptable to climatic conditions and dry soils [8] due to the contribution of a high amount of nutrients to the soil, protecting them from external factors such as erosion, desiccation, and high temperatures [4], with an optimum growth temperature of 25–35 °C, but being able to withstand even 48 °C for a limited time [9]. Regarding rainfall, it withstands drought well, with its optimum range being around 1000 mm per year; however, it should be noted that at values below 250 mm, leaf production is seriously affected [10]. The agronomic and nutritional characteristics of *M. oleifera* could help against the effects of climate change [11], which is expected to continue to increase temperatures and alter precipitation patterns [12]. Due to these changes, water consumption in agriculture will increase, and new transformations in the sector will be necessary, such as in the Mediterranean basin, which is a geographical area highly exposed to the effects of this process, as indicated by different recent studies [13]. The convergence in this geographical space of high climatic hazard and the intense occupation of the territory, especially in coastal strips, make Mediterranean regions areas of high risk, which has increased in recent decades [14]. Therefore, the climatological and agronomic adaptability of this tree makes it a perfect crop for development in the Iberian Peninsula and the Balearic Islands, with special emphasis on the Andalusian coast, the Valencian and Murcian coasts, and other small locations on the Mediterranean coast, such as the Ebro Delta basin [15].

All parts of the moringa can be considered edible and have a multitude of uses [16]. In the specific case of its seeds, the extraction of the oil can be a good alternative to other vegetable oils, diversifying the current market. Thus, it could respond to problems such as the shortage of sunflower oil in Western Europe due to the lack of fertilizers, the exponential increase in fuel prices, and problems with the food supply logistics system due to the blockade of Ukrainian ports in the Black Sea [17].

The seeds of this plant are round, with a dark brown color, three whitish wings, and a thin membrane surrounding them. The plant produces a variable number of seeds per pod, but it is estimated that a tree can produce between 15,000 and 25,000 seeds annually [6,18]. It stands out for its use as one of the most important natural flocculants commercially available for water treatment, including residual water [19], while the flour extracted from the seeds is usually used for animal feed.

Some studies highlight the high antioxidant activity of moringa seeds and have isolated a large number of phytochemical compounds that can be used as nutraceutical bioactive molecules, capable of reducing oxidative damage associated with aging and cancer and may also be potential antitumor promoters [5,20,21].

Moringa seeds also contain an oil percentage of 19–40%, with an intense yellow color and low viscosity. It is rich in monounsaturated and polyunsaturated fatty acids, like oleic acid (C18:1 ω9) and linolenic acid (C18:3 ω3), with oleic acid being the main fatty acid, accounting for approximately 70%. Moreover, linolenic acid (ω3) is an essential fatty acid, which means that the human body cannot synthesize it; thus, it must be obtained throughout the diet. ω3 fatty acids are associated with pro-inflammatory responses, and ω9 fatty acids serve as necessary components for other metabolic pathways, which may affect disease risk [22]. In addition, it contains other fatty acids, such as behenic, palmitic, and stearic acids.

It also has a large number of tocopherols, such as vitamin E, which is why it can be used as a good and effective supplement in reducing cholesterol [23]. For this reason, it is usually added to other oils to obtain mixtures with high nutritional properties [24,25]. In this sense, it can be used as a culinary substitute for other vegetable oils. Other interesting industrial uses would be the production of cosmetics, lubricants, and biodiesel [26,27].

In the current context, the search for more sustainable food production alternatives is necessary to reduce negative effects on the environment. One possibility would be to promote the production of moringa since it is one of the crops that requires high temperatures for development and requires little water input. The Spanish Mediterranean basin is a region that meets the requirements for moringa to be cultivated successfully [15].

The extraction of oil from the moringa seed has traditionally been carried out using the Soxhlet technique and with the use of solvents, generally n-hexane [28,29,30]. Cold pressing has also been used, but it reduces the oil extraction yield; only 69% of the total oil contained in the moringa seed can be extracted via cold pressing [31,32,33].

To our knowledge, the characterization of moringa seed oil extracted at different temperatures has not been reported. In addition, most of the studies published are focused on extraction with solvents, which would imply that these oils are not suitable for food use unless they are submitted to a refined process. For this reason, the aim of this study is to analyze the amount of fatty acids along with other quality properties of moringa oil using an extraction system appropriate for the food industry at different temperatures.

## 2. Materials and Methods

### 2.1. Raw Material

For oil extraction, moringa seeds from pods of trees grown in an experimental plot of the Universitat Politècnica de València (Valencia, Spain) located at Camino de Vera s/n (39°29′02.2 N 0°20′09.6 W) were used as raw materials. Specifically, the trees were planted in 2016, and the pods were harvested between March and April 2021. The pods were stored for approximately one month in a dry place before obtaining the seeds to favor water removal. The moisture content of the seeds was 4.5 ± 0.2%, which was determined via the gravimetric method adapted from AOAC 930.15 [34]. For this purpose, the samples were pre-dried in an oven at 60 °C for 24 h and then dried in a vacuum oven (J.P SELECTA, Conterm model, Barcelona, Spain) at 60 °C for 48 h until a constant weight was reached.

The seeds were then dehulled with an electric shredder (Centrale Brico, YT542 230 V, Lognes, France) and passed through a 4 mm mesh sieve to remove as many impurities as possible.

### 2.2. Moringa Oil Extraction and Yield

An automatic press extractor (Cgoldenwall 350 W 3–6 kg/h, Madrid, Spain) was used, consisting of a worm screw that operates at a temperature range of 20–220 °C. The following extraction temperatures were used in this study: 70, 100, 130, 160, 190, and 220 °C. The extraction process was carried out from approximately 100 g of seeds for each of the 3 replicates per temperature. The oil yield was expressed in percentage with respect to the initial seed mass in triplicate for each temperature.

### 2.3. Optical Properties of the Oil

CIEL*a*b* coordinates were determined in the oil using a spectrocolorimeter (“Konica Minolta” Inc. Model CM—3600d, Tokyo, Japan) with a D65 reference illuminant and a 10° observer. With the aim to analyze its reflectance, the color was measured using a white and a black background to obtain the Kubelka–Munk coefficient [35,36,37]. There were 6 replicates per treatment.

### 2.4. Determination of Chemical Characteristics

Acidity (A) and acid values (AVs) were determined according to UNE-EN ISO 660:2010 [38] for oils and fats of animal and vegetable origin. For this purpose, 5 g of oil was weighed in a 250 mL Erlenmeyer flask. Then, 12.5 mL of 96° ethyl alcohol (Panreac AppliChem ITW reagents, Barcelona, Spain) and 12.5 mL ethyl ether AGR stabilized with BHT (LabKem, Barcelona, Spain) were added. After that, 1 mL of 1% phenolphthalein (LabKem, Barcelona, Spain) was added. The mixture was gently stirred until it became a homogeneous solution. A manual titration was then carried out with a 0.1 M KOH solution (Scharlau, Barcelona, Spain) until the color turned from yellow to pink. To calculate the percentage of free fatty acids (A) and the acid value (AV), the following equations were used:

(1) $$A\left(\frac{g\;oleic\;acid}{100\;g\;oil}\right)=100\cdot \frac{282\cdot V\cdot N}{W}$$

(2) $$AV\left(\frac{mL\;KOH}{g\;oil}\right)=1.99\cdot A$$

where

V = volume of KOH consumed in the titration of oil sample (mL);
N = normality of titration solution (KOH);
W = weight of oil sample used (g).

To evaluate the iodine value (IV), official Panreac analysis methods for oils and fats [39] were followed, and 0.3 g of an oil sample was weighed in an Erlenmeyer flask and mixed with 10 mL of chloroform (Honeywell Riedel-de Haën, Barcelona, Spain), and the mixture was stirred to dissolve it. Then, 25 mL of Hannus reagent (Carlo Erba, Dasit Group, Sabadell, Spain) was added, and the mixture was gently stirred. Next, the mixture was covered and left in the dark for 60 min. After this time, 20 mL of 10% potassium iodide (LabKem, Barcelona, Spain) was added along with 100 mL of distilled water and two drops of 1% (*w*/*v*) starch solution (Scharlau, Barcelona, Spain) as an indicator was also added, and the mixture was softly mixed once more. The titration was then carried out with 0.5 N Na_2_S_2_O_3_ (LabKem, Barcelona, Spain) until the solution acquired a transparent whitish color. A blank was made, which contained all of the aforementioned chemicals, apart from the sample. The iodine value was obtained with the following equation:

(3) $$IV\left(\frac{g{\;I}_{2}}{100\;g\;oil}\right)=\frac{0.1269\cdot N\cdot (V_{B}-V_{S})}{W}$$

where

V_B_ = volume of Na_2_S_2_O_3_ consumed in the titration of blank solution (mL);
Vs = volume of Na_2_S_2_O_3_ consumed in the titration of oil sample (mL);
N = normality of Na_2_S_2_O_3_ solution;
W = weight of oil sample used (g).

The saponification value (SV) was determined by dissolving 2 g of the oil in 25 mL of 0.5 M ethanolic KOH in a 250 mL flask. The mixture was heated in a water bath at 100 °C for half an hour. Then, two drops of phenolphthalein indicator were added to the content of the flask to be titrated afterward with 0.5 N HCl (Panreac AppliChem 37%, Barcelona, Spain). A blank determination was also carried out under the same conditions but without oil. The SV was calculated using the following equation:

(4) $$SV\left(\frac{mg\;KOH}{g\;oil}\right)=\frac{56.1\cdot N\cdot (V_{B}-V_{S})}{W}$$

where

V_B_ = volume of KOH consumed in the titration of blank solution (mL);
V_s_ = volume of KOH consumed in the titration of the oil sample (mL);
N = normality of HCl solution;
W = weight of oil sample used (g).

### 2.5. Determination of Oxidative Stability

The K_232_ and K_270_ specific extinction coefficients were determined according to UNE 55.047–73 by weighing 0.2 g of oil, adding 10 mL of cyclohexane for HPLC > 99.7% (Honeywell Riedel-de Haën, Barcelona, Spain), and measuring the absorbance at wavelengths of 232 and 270 nm. The following equation was used to calculate these parameters:

(5) $$K_{232}\;or\;K_{270}=\frac{A}{P\cdot l}$$

where “A” is the absorbance read on the spectrophotometer (Thermo Fisher Scientific, Inc. Helios Zeta UV-VIS, Waltham, MA, USA), “l” is the thickness of the cuvette in cm, and “P” is the weight of the sample in mg.

To obtain the peroxide value (PV), a titration was performed with an automatic titrator (Metrohm with Titrando 905, Ti Stand 804 modules, and platinum electrode, Madrid, Spain) using 0.001 N Na_2_S_2_O_3_ as a titrant solution in moringa oils stored for 1 year in glass dark jars at room temperature. To carry out the titration, 1 g of the sample was weighed, and 10 mL of the solvent composed of acetic acid (J.T. Baker, Madrid, Spain) and 7.5% I_2_ (Iodine resublimed extrapure Scharlau, Barcelona, Spain) in 1-decanol 98% (Thermo Scientific) in a ratio of 3:2. Subsequently, 200 µL of the saturated KI (LabKem, Barcelona, Spain) solution was added, allowing this mixture to stand in the dark for 1 min. After this time, 50 mL of boiled distilled water was added. Finally, the mixture was titrated in the automatic titrator, obtaining the results in milliequivalents of O_2_/kg of oil.

### 2.6. Determination of Antioxidant Capacity (AC)

The radical scavenging activity of moringa oils was determined using the DPPH method according to the method used in [40]. For this purpose, 3.9 mL of a solution of 0.024 g/L of 1,1-diphenyl-2-picrylhydrazyl (Sigma-Aldrich, Darmstadt, Germany) was prepared and mixed with 100 µL of each oil, and the mixture was incubated for 30 min in the dark. After that, the absorbance was measured at 515 nm on the spectrophotometer (Thermo Fisher Scientific, Inc. Helios Zeta UV-VIS, Waltham, MA, USA).

The sample activity of the scavenging DPPH radicals was calculated as follows:

$$AC\left(\%\;Inhibition\;DPPH\right)=100\cdot \frac{Absorbance\;of\;control-Absorbance\;of\;sample}{Absorbance\;of\;control}$$

### 2.7. Fatty Acids Profile of Moringa Oil

The fatty acid compositions were determined according to the International Olive Council [41]. Fatty acids were transesterified into their corresponding fatty acid methyl esters (FAMEs) by shaking a solution of 0.1 g of oil and 2 mL of n-hexane 96% (Scharlau, Barcelona, Spain) with 0.2 mL of 2 N methanolic KOH. The FAMEs were then analyzed via gas chromatography (Agilent Technologies, 7820A GC System, Madrid, Spain) equipped with a flame ionization detector (FID) using a fused-silica capillary column (DB-23 Agilent Technologies, 60 m × 0.25 mm internal diameter 0.2 µm film thickness). Both the injector and the detector temperatures were held at 250 °C. For the analysis, the oven was heated using 5 ramps. First, a heating ramp of 25 °C/min was applied from 50 to 185 °C. In the second run, it reached 190 °C at a rate of 4 °C/min, maintaining this temperature for 1.5 min. This heating rate of 4 °C/min was used in the following ramps. It then rose to 195 °C, remaining at this temperature for 5 min. Then, it went up to 200 °C and remained at this temperature for 1.5 min. Finally, it reached 230 °C and was maintained at this temperature for 5 min. This oven temperature program is summarized in Table 1.

The injected volume of the FAMEs was 1 µL, and helium was used as the carrier gas at 1 mL/min. Fatty acids were identified by comparing retention times with those of standard compounds (F.A.M.E. mix C14-C22 certified reference material ampule 100 mg, Sigma Aldrich, St. Louis, MO, USA).

### 2.8. Statistical Analysis

Statgraphics Centurion (XIX.64) software was used to statistically analyze the results. An ANOVA analysis of variance was performed using the LSD (least significant difference) test at a significance level of 95% (*p*-value ≤ 0.05).

## 3. Results and Discussion

### 3.1. Moringa Seed Oil Yield

**Figure 1:**
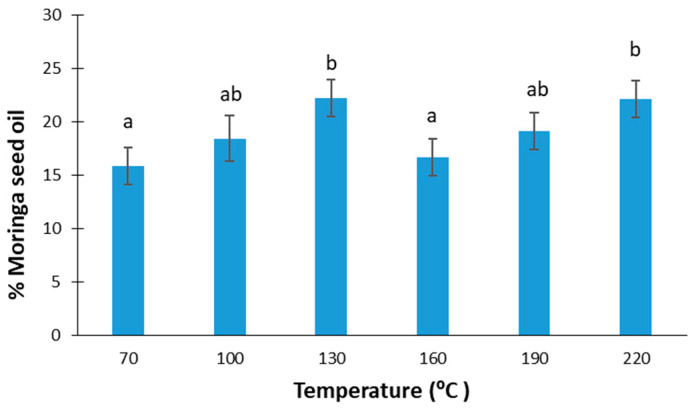
Moringa seed oil yield at different extraction temperatures. Equal lowercase letters refer to similar homogenous groups with a significance level of 95%.

Figure 1 shows the percentages of oil extracted in an automatic press at different temperatures from *Moringa oleifera* seeds. According to the ANOVA performed, no significant differences were observed in the oil yield as a function of the temperature applied (F-ratio: 1.52; *p*-value: 0.2543), with the mean value obtained being 19 ± 3%. According to [24], moringa oil extraction at 100 °C with chloroform/methanol (C/M) in a 3:1 ratio had the highest yield (41%) in comparison with other combinations of C/M, and other temperatures in the range of 70–110 °C. On the other hand, in the case of Soxhlet extraction using different solvents, like petroleum ether, n-hexane, or isopropyl alcohol, an oil extraction of around 25.5–43.5% was achieved [28,29,30,41,42,43].

Considering that moringa seeds can contain between 19 and 47% oil [24], the oil recovery percentage can be at most 58%. However, in other studies, where moringa oil was extracted with solvents, such as petroleum ether solvent, the recovery percentage was 90%; however, if ultrasound or microwaves were also applied, it could reach up to 91–94% [44].

### 3.2. Optical Properties

**Figure 2:**
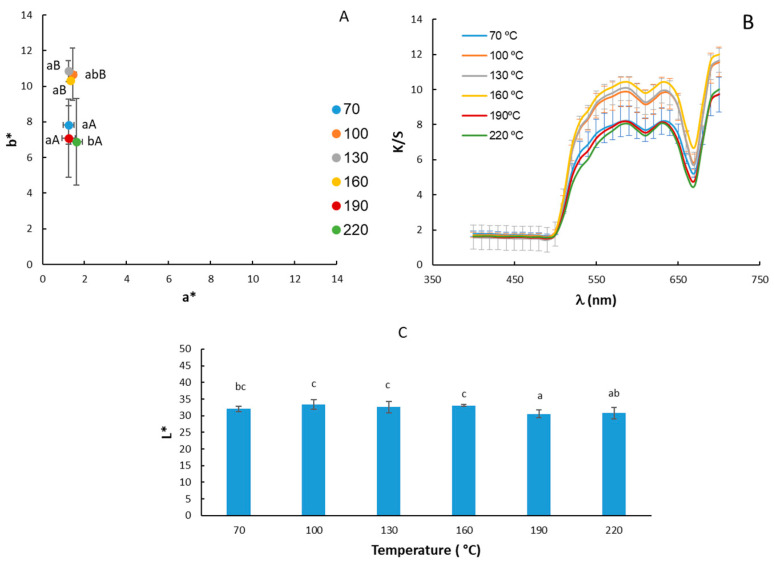
Location on the chromatic plane of the coordinates a* and b* (**A**); values of the Kubelka–Munk (K/S) coefficient (**B**) and luminosity L* (**C**) of moringa oil extracted at different temperatures. The same letters indicate homogeneous groups obtained in the ANOVA, with a significance level of 95%, considering the temperature factor. Lowercase letters are used for the a* coordinate and uppercase letters are used for the b* coordinate.

The results of the color determinations of moringa oil are shown in Figure 2. Regarding the chromatic diagram of the a* and b* coordinates as a function of the oil extraction temperature (Figure 2A), the samples were located in the first quadrant with higher b* values, between 6 and 12. On the other hand, the average values of the a* coordinate were close to 1, that is, in brownish-yellow tones. The results obtained for the a* coordinate match those obtained by [45]. On the other hand, those obtained for the b* coordinate are lower in our study (8.95 ± 1.9) compared to the value of (64 ± 2) obtained by [45], indicating less yellow and darker tones compared to those obtained via cold pressing, which is more yellow. The statistical analysis showed significant differences both for the a* coordinate (*p*-value: 0.03749) and, especially for the b* coordinate (*p*-value: 0.000) with respect to the extraction temperature factor. However, a much lower b* value was only observed when high temperatures (190 and 220 °C) were used in the oil extraction, although for 70 °C, the b* values were similar to these cases. These results would be consistent with the pigment profile of cold-pressed oils, in which carotenoids and chlorophylls predominate [46].

The lightness values of moringa oils ranged from 30.5 to 33.4. No major changes were observed in the lightness of the samples according to the extraction temperature (Figure 2C). The lightness obtained in the present study is about half that obtained by Athikomkulchai et al., 2021 [45], for moringa oil obtained via cold pressing.

The K/S coefficient values (Figure 2B) were lower in oils extracted at higher temperatures 160, 190, and 220 °C, except for the oil extracted at 70 °C. Therefore, the increase in extraction temperature would lead to lower oil transparency [37].

### 3.3. Chemical Characteristics

The results obtained relating to the chemical properties of moringa oil extracted at different temperatures are shown in Table 2.

The acidity of the oil provides information on the percentage of the free fatty acids it contains. This parameter is one of the chemical characteristics that best defines the quality of an oil as it represents the hydrolytic deterioration to which it has been subjected. In this case, the acidity of moringa oil samples analyzed in this study was approximately 2 g oleic acid/100 g oil. This value fulfills the acidity regulations of Codex Alimentarius (2021) required for virgin olive oil (≤2 g oleic acid/100 g oil) [47]. Therefore, moringa oil is suitable and of adequate quality for human consumption. The acidity obtained in this study was slightly higher than that obtained by [48] for moringa seed oil obtained via cold pressing; they obtained an acidity value of 0.19 g oleic acid/100 g oil. On the other hand, the acid value of the studied samples ranged between 3 and 3.6 mg KOH/g oil. These results are in accordance with those previously reported by [29], who obtained an acid value of 4.21 mg KOH/g oil for *Moringa oleifera* seed oil. Having such a high free fatty acids content, soap formation may occur during the alcoholysis process from its by-products.

The iodine value (IV) provides quantitative information on the presence of unsaturated fats and oils. The higher iodine value indicates that the double bonds present in the fatty acids of the oil are prone to react with halogens, such as iodine. The IV of the analyzed samples of moringa oil ranged between 124 and 168 g I_2_/100 g oil without statistically significant differences. These values were higher than those obtained by other authors for moringa seed oil [30,45,49,50], who obtained iodine values between 67 and 85 g I_2_/100 g oil. Differences in these parameters may be due to the variety of moringa, the geographical origin or the climatic conditions where it has been cultivated, as other authors have reported for olive oil [51]. The values obtained in this study for iodine value also are slightly higher than that established by the Codex Alimentarius [47] for olive oil (75–94 g I_2_/100 g oil), which means that most of the fatty acids present in moringa seed oil are unsaturated.

The saponification value (SV) is a measure of the total amount of fatty acids (both free and esterified in the form of triglycerides). It provides information on the average molecular weight of all fatty acids present in the oil. The higher the SV, the lower the molecular weight of all fatty acids. In this study, the SV of the analyzed moringa oils ranged between 187 and 219 mg KOH/g oil. A gentle decrease was observed with increasing oil extraction temperature. These values were slightly higher than those established by legislation (for olive oil (184–196 mg KOH/g oil) [47]. The results of saponification obtained in this study were subtlety higher than those reported by [52] (168.3 ± 0.45 mg KOH/g oil) for the Malaysian Moringa seed oil extracted using petroleum ether but similar to those reported by [33] for cold-pressed Indian *Moringa oleifera* seed oil (190.4 mg KOH/g oil), as well as those extracted using hexane (191.2 mg KOH/g oil). Furthermore, [45] obtained similar results to ours for the saponification value of moringa seed oil obtained via cold pressing (187 mg KOH/g oil).

Last but not least, the antioxidant capacity (AC) of moringa oil was similar to those reported by other authors [30]. No changes in AC were observed due to the temperature of the extraction, except for the highest temperature, where there was a decrease. This AC could be related to the content of α-tocopherol present in moringa oil [44].

### 3.4. Oxidative Stability of Moringa Seed Oils

The oxidative state of moringa seed oil was determined by measuring the peroxide value and specific extinction coefficients K_232_ y K_270_ after one year of storage. The results are shown in Table 3. The oxidation of oil occurs when oxygen is incorporated into an unsaturated fatty acid. As soon as oil begins to oxidize, various compounds form. These include peroxides, which are considered the main oxidation products. The PV determines the initial oxidation states of oil and the deterioration that natural antioxidants, polyphenols, and certain components of nutritional interest, such as vitamin E and tocopherols, may have suffered. In this study, the PV ranged from 0.95 to 3 meqO_2_/kg oil, increasing with oil extraction temperature. In all cases, these values are below the levels established by the legislation for virgin olive oil, which establishes that the peroxide value must be less than 20 meqO_2_/kg [47]. This low value obtained in terms of PV makes this oil suitable for long storage periods due to the low level of oxidative activity. These very low results are in line with the PV values reported by other authors for moringa oil. Ref. [53] reported a 0.19 ± 0.12 meqO_2_/kg oil peroxide value for moringa oil obtained via cold pressing and [48] reported a value of 0.49 meqO_2_/kg oil.

The specific extinction coefficients K_232_ y K_270_ provide information on the quality of an oil and its state of preservation. K_232_ provides information on the early stages of oxidation, while the K_270_ parameter is an indicator of advanced oxidation phases (secondary oxidation). Some of these secondary oxidation products are volatile and contribute to the noticeable presence of a rancid aroma. A high absorption rate at 270 nm is related to oxidation in virgin oil and/or the refining process. The analyzed oils did not exceed the value of 2.50 for K_232_ or 0.22 for K_270_, established as the limits for olive oil [47]. The values obtained in this work for K_270_ and K_232_ are lower than those obtained by other authors for moringa oil [31,50], which indicates that the moringa oil samples have low levels of both primary and secondary oxidation.

### 3.5. Fatty Acid Profile of Moringa Seed Oils

**Figure 3:**
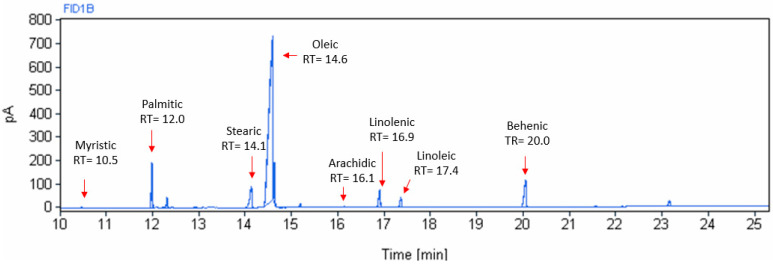
Chromatographic spectrum of fatty acids of moringa seed oil extracted at 70 °C.

Figure 3 shows a typical chromatogram of moringa oil, where the different peaks and their retention time of the fatty acids present in moringa oil can be observed. As expected, the largest peak corresponds to oleic acid.

The fatty acid profiles of the moringa seed oil extracted at different temperatures, expressed as the mean percentage value of each fatty acid with respect to the total fat content, are presented in Table 4. Eight fatty acids were detected and identified with an increasing number of carbons, as follows: myristic acid (C14:0), palmitic acid (C16:0), stearic acid (C18:0), oleic acid (C18:1 ω9), linoleic acid (C18:2 ω6), linolenic acid (C18:3 ω3), arachidic acid (C20:0), and behenic acid (C22:0).

These results suggest that the total monounsaturated fatty acid (MUFA) content fluctuated between 77.34 and 77.82%, with oleic acid (C18:1 ω9) being the major fatty acid present in the oil. These values are coherent with those obtained with other techniques of extraction, like cold press (71.6% or 75.5 ± 0.7%) [48,54] or Soxhlet extraction (73.8 ± 0.7%) [54]. However, in our case, the amount of oleic acid was higher than that reported by [44], who extracted moringa oil using conventional Soxhlet (68.3 ± 0.3%) or combined with microwaves (68.14 ± 1.03%), ultrasound (66.3 ± 0.6%), or CO_2_ (69.7 ± 0.3%). Therefore, the extraction method applied in this study does not seem to reduce the value of the main fatty acid of moringa, which is oleic acid.

Oleic acid may have favorable nutritional implications and may substantially contribute to the prevention of both cardiovascular disease and cancer [55]. 

On the other hand, the polyunsaturated fatty acid (PUFA) content ranges from 3.69 to 3.99%, with linolenic (C18:3 ω3) being the main one, with a concentration between 3.215 and 3.46%. In addition, linoleic acid (C18:2 ω6) was found at a lower proportion, between 0.3 and 0.55%. On the subject of unsaturated acids, they contribute to the maintenance of normal blood cholesterol levels, and functional foods high in oleic acid have been supported by related health claims in the EU [56].

In regard to total saturated fatty acids (SFAs), they accounted for 18% approximately. Behenic acid (up to 6%) was found to be the dominant saturated fatty acid, followed closely by palmitic acid (C16:0) and stearic acid (C18:0). According to [57], behenic acid has a low bioavailability, which reduces absorption and results in dwindling body weight and the deposition of visceral fat [58].

Arachidic acid was found in a concentration ranging from 0.19 to 0.225%. Finally, the minor fatty acid was myristic (C14:0), with a concentration of 0.09%. These results fall within the range of those reported by other authors for moringa oil [29,31,47]. However, the fatty acid profiles may vary slightly depending on the moringa variety and the oil extraction method used [54].

No statistically significant differences were observed in either oleic acid (monounsaturated) or linoleic acid (polyunsaturated) depending on the extraction temperature. These results are in accordance with [59], who reported that oleic acid in olive oil was not affected by the oil extraction temperature.

On the other hand, a decrease was observed in linolenic acid (polyunsaturated), starting at a 130 °C extraction temperature. This result is consistent with [30], who observed a decreasing trend in linoleic acid, which might be due to the preferential cleavage of double bounds.

Regarding saturated fatty acids, an increase in palmitic and behenic acid was observed at 160 °C and above. On the other hand, myristic, stearic, and arachidic acids showed a decreasing tendency when the extraction was carried out at a temperature higher than 130 °C. In comparison with the results relating to behenic acid obtained by [54] and [32] using cold extraction (5.83 ± 0.13% and 6.2 ± 0.5%, respectively), our extraction methodology improved the percentage of this fatty acid. On the other hand, the values of the stearic acids were quite similar to those reported by [32] (5.7 ± 0.2%), although a bit higher than in the case of [54] (2.81 ± 0.13%). For palmitic acid, our values were lower than the values obtained by [32,54] (6.3 ± 0.4% and 9.6 ± 0.3%, respectively).

This fatty acid composition of *Moringa oleifera* seed oil falls into the category of high oleic oils and contains a high proportion of monounsaturated to saturated fatty acids (MUFA/SFA). The high MUFA/SFA ratio is characteristic of several oils, particularly olive oil, and has been associated with a reduced risk of all-cause mortality, cardiovascular mortality, cardiovascular events, and stroke [60]. 

Despite the fact that the PUFA/SFA ratio has been traditionally used to evaluate the cardiovascular disease-preventive characteristics of fats, this ratio excludes MUFA, including oleic acid, which is a major component of moringa oil and is known to greatly contribute to combating these diseases [61]. This is why, in this work, the USFA/SFA ratio was used, obtaining values of around 4.4. The results of this study are lower than those values obtained by other authors in relation to moringa oil (≈6) [30].

*Moringa oleifera* seed oil has a monounsaturated fatty acid content similar to that of olive oil but a lower polyunsaturated fatty acid content [30,34]. Therefore, this moringa oil could be an acceptable alternative to olive oil since its fatty acid profile is very similar.

## 4. Conclusions

The extraction temperature of the oil did not affect the yield or the main quality characteristics of the oil. However, the oils extracted at temperatures higher than 130 °C showed an increased peroxide index, evidencing higher oxidation but within the limit allowed by olive oil legislation. Oleic acid (≈77%) is the predominant fatty acid in moringa oil, with a content similar to that of olive oil. In addition, it has approximately 7% behenic acid, 6% palmitic acid, 5% stearic acid, and 3% linolenic acid. This composition implies a USFA/SFA ratio similar to that of other vegetable oils that are considered suitable for the prevention of cardiovascular disease. In addition, it is remarkable that behenic acid is not found in most vegetable oils; furthermore, despite being a saturated fatty acid, it has been related to the prevention of obesity due to its low bioavailability. In terms of practical applications, moringa oil has been extracted using an eco-friendly and affordable method that could be extrapolated to the food industry. For future perspectives, assays focusing on bioactivity would be performed. All things considered, the good properties of moringa oil make it a good, sustainable alternative to vegetable oils.

## Figures and Tables

**Table 1:** Heating ramps of temperature GC-FID.

	Rate (°C/min)	Temperature (°C)	Hold Time (min)	Run Time (min)
Ramp 1	25	185	0	6.4
Ramp 2	4	190	1.5	9.15
Ramp 3	4	195	1.5	11.9
Ramp 4	4	200	1.5	14.65
Ramp 5	4	230	5	27.15

**Table 2:** Chemical properties of moringa seed oil extracted at different temperatures.

	Moringa Seed Oil Extracted at Different Temperatures (°C)						
	70	100	130	160	190	220	F-Ratio
A ^1^	1.8 ± 0.4 ^a^	2.06 ± 0.09 ^a^	2.09 ± 0.14 ^a^	2.13 ± 0.11 ^a^	1.9 ± 0.5 ^a^	2.1 ± 0.08 ^a^	0.02
AV ^2^	3.6 ± 0.9 ^a^	4.1 ± 0.2 ^a^	4.2 ± 0.3 ^a^	4.2 ± 0.2 ^a^	3.8 ± 1 ^a^	4.2 ± 1.2 ^a^	0.02
SV ^3^	219 ± 3 ^b^	198 ± 9 ^ab^	199 ± 2 ^ab^	196 ± 4 ^ab^	187 ± 7 ^a^	199 ± 2 ^ab^	1.79
IV ^4^	130 ± 40 ^a^	155 ± 1.5 ^a^	168 ± 1.5 ^a^	124 ± 22^a^	126 ± 43 ^a^	144 ± 18 ^a^	0.89
AC ^5^	62.2 ± 0.7 ^b^	63 ± 2 ^b^	61.9 ± 1.6 ^b^	61.9 ± 0.7 ^b^	63 ± 3 ^b^	57.9 ± 0.9 ^a^	2.86

Values with different letters are significantly different, with a significance level of 95%. ^1^ Acidity (g oleic acid/100 g oil); ^2^ acid value (mg KOH/g oil), ^3^ Saponification value (mg KOH/g oil); ^4^ iodine value (g I_2_ 100 g oil); ^5^ antioxidant capacity DDPH (% inhibition).

**Table 3:** Determination of the oxidative state of the moringa seed oil extracted at different temperatures after one year of storage.

	Moringa Seed Oil Extracted at Different Temperatures (°C)						
	70	100	130	160	190	220	F-Ratio
^1^ PV	0.95 ± 0.3 ^a^	0.99 ± 1.1 ^a^	1.3 ± 0.3 ^ab^	3.1 ± 0.9 ^c^	2.2 ± 0.3 ^bc^	3.0 ± 0.3 ^c^	7.05 **
10^4^·K_232_	270 ± 3 ^a^	270 ± 4 ^a^	290 ± 4 ^b^	290 ± 6 ^b^	300 ± 3 ^d^	300 ± 0.6 ^c^	119.85 **
10^4^·K_270_	23.46 ± 0.08 ^e^	10.3 ± 1.2 ^a^	18.8 ± 0.5 ^d^	14.86 ± 0.09 ^b^	14.20 ± 0.18 ^b^	17.1 ± 0.2 ^c^	274.52 **

Values with different letters are significantly different: ** 99% significance level. ^1^ Peroxide value expressed as meqO_2_/kg oil.

**Table 4:** Fatty acid (%) composition of Moringa oleifera seed oil extracted at different temperatures.

	Temperature (°C)						
	70	100	130	160	190	220	F-Ratio
1	0.095 ± 0.003 ^a^	0.095 ± 0.004 ^a^	0.092 ± 0.002 ^b^	0.09 ± 0.003 ^bc^	0.09 ± 0.0018 ^bc^	0.0893 ± 0.0019 ^c^	8.17 **
2	5.88 ± 0.07 ^a^	6.02 ± 0.13 ^a^	5.92 ± 0.06 ^a^	6.3 ± 0.4 ^b^	6.50 ± 0.06 ^b^	6.48 ± 0.08 ^b^	20.96 **
3	5.38 ± 0.02 ^a^	5.39 ± 0.05 ^a^	5.42 ± 0.03 ^a^	5.0 ± 0.3 ^b^	4.820 ± 0.016 ^c^	4.90 ± 0.03 ^c^	42.68 **
4	77.8 ± 0.4 ^b^	77.3 ± 0.3 ^a^	77.6 ± 0.3 ^ab^	77.5 ± 0.3 ^ab^	77.7 ± 0.4 ^ab^	77.4 ± 0.4 ^a^	2.45 *
5	0.3 ± 0.4 ^a^	0.536 ± 0.006 ^b^	0.53 ± 0.01 ^b^	0.49 ± 0.05 ^b^	0.476 ± 0.002 ^b^	0.491 ± 0.009 ^b^	0.55
6	3.440 ± 0.018 ^c^	3.456 ± 0.019 ^c^	3.46 ± 0.02 ^c^	3.312 ± 0.012 ^b^	3.215 ± 0.012 ^a^	3.280 ± 0.011 ^b^	37.34 **
7	0.225 ± 0.005 ^a^	0.221 ± 0.005 ^a^	0.221 ± 0.005 ^a^	0.201 ± 0.018 ^bc^	0.19 ± 0.03 ^c^	0.206 ± 0.003 ^b^	20.33 **
8	6.84 ± 0.12 ^a^	6.9 ± 0.1 ^bc^	6.80 ± 0.05 ^a^	6.97 ± 0.15 ^cd^	7.03 ± 0.07 ^d^	7.20 ± 0.05 ^e^	19.33 **
SFA	18.42	18.66	18.44	18.65	18.64	18.87	
MUFA	77.82	77.34	77.57	77.53	77.66	77.35	
PUFA	3.76	3.99	3.99	3.80	3.69	3.77	
USFA	81.6	81.3	81.6	81.3	81.4	81.1	
USFA/SFA	4.43	4.36	4.42	4.36	4.37	4.30	
SFA/USFA	0.226	0.229	0.226	0.229	0.229	0.233	

SFAs: saturated fatty acids; MUFAs: monounsaturated fatty acids; PUFAs: polyunsaturated fatty acids; USFAs: unsaturated fatty acids. Values are means ± SD of three determinations. Values with different letters are significantly different: * 95% significance level, ** 99% significance level. 1: Myristic (C14:0), 2: palmitic (C16:0), 3: stearic (C18:0), 4: oleic (C18:1 ω9), 5: linoleic (C18:2 ω6), 6: linolenic (C18:3 ω3), 7: arachidic (C20:0), and 8: behenic (C22:0).

## Data Availability

The original contributions presented in the study are included in the article, further inquiries can be directed to the corresponding author.
